# Calendar

**DOI:** 10.1155/S1463924686000421

**Published:** 1986


					Journal of Automatic Chemistry/Journal ofClinical Laboratory Automation, Vol. 8, No. 4 (October-December 1986), p. 215
Calendar
MAY
13 to 15 May Scientific Computing and Automation (Europe):
Amsterdam, The Netherlands. Contact Scientific Computing
and Automation (Europe), c/o Reunion International
BV, Tholenseweg 3, 1181 KD Amstelveen, The Nether-
lands.
OCTOBER
22 and 23 October Seminar: CrossJlow Membrane Filtration:
Minnetonka, Minnesota, USA. Contact Laura Wedding,
OSMO Membrane Systems Division, OSMONICS,
Inc., 5951 Clearwater Drive, Minnetonka, Minnesota
55343, USA.
JUNE
28 June to 4July 13th Annual Congress on Clinical Chemistry:
The Hague, The Netherlands. Contact Dr N. C. den Boer,
PO Box 82000, 2508 EA, The Hague, The Netherlands.
23 to 25 October Annual Joint Meeting of the German,
Austrian andSwiss Societies ofClinical Chemistry with the Socigtg
Franfaise de Biol. Clinique: Basel, Switzerland. Contact Dr
Peter R. Huber, Laboratorien FK, Kantonsspital Basel,
CH 4031 Basel, Switzerland.
JULY
13 to 18 July 31st International Congress ofPure and Applied
Chemistry: Sofia, Bulgaria. Contact Dr R. Vlahov, Institute
of Organic Chemistry, Bulgarian Academy of Sciences,
1i 13 Sofia, Bulgaria.
NOVEMBER
10 to 12 November International Symposium on New Sensors
and Methodsfor Environmental Characterization: Kyoto Japan.
Contact Professor M. Matsui, Institute for Chemical
Research, Kyoto University, Uiji, Kyoto 611, Japan.
19 to 24 July Annual Meeting ofthe American Association of
Clinical Chemistry: San Francisco, California, USA. Contact
AACC, Office ofContinuing Medical Education, 1725 K
Street N.W., Washington, D.C. 20006.
SEPTEMBER
11 to 12 November Symposium on Control and Prevention of
Runaway Chemical Reaction Hazards: Hotel Okura, Amsterdam,
The Netherlands. Contact Fiona Spindlove, IBC Technical
Services Ltd, Bath House, 56 Holborn Viaduct, London
EC 1A 2EX.
4 to 6 September IMLS: Quality Assurance Symposium:
Liverpool, UK. Contact Mr D. Kilshaw, Department of
Pathology, Arrowe Park Hospital, Arrowe Park Road,
Upton, Wirral, Merseyside L49 5LN, UK.
1988
1987 APRIL
MARCH
9 to 13 March 38th Pittsburgh Conference and Exposition on
Analytical Chemistry and Applied Spectroscopy: Convention
Center, Atlantic City, New Jersey, USA. Contact Mrs Alma
Johnson, Program Secretary, 12 Federal Drive, Suite 322,
Pittsburgh, Pennsylvania 15235, USA.
16 to 21 April 1988 American Society for Clinical Pathology
Spring National Meeting: Kansas City, USA. Contact ASCP,
2100 West Harrison, Chicago, Illinois 60612-37798,
USA.
18 to 21 April Flow Analysis IV: Las Vegas, Nevada, USA.
Contact Professor Gilbert Pacey, Department of Chem-
istry, Miami University, Oxford, Ohio 45056, USA.
APRIL
3 to 5 April IMLS: Clinical Chemistry/Haematology/Blood
Group Serology Symposium: York, UK. Contact
Mr K. V. Lewis, Pathology Department, Royal Infirm-
ary, Bradford, West Yorkshire BD9 6RJ, UK.
6 to 9 April International Symposium on Electroanalysis in
Biomedical, Environmental and Industrial Sciences: Cardiff
UK. Contact Short Courses Section, UWIST, PO Box 68,
Cardiff CF1 3XA, UK.
JULY
24 to 29 July National Meeting ofthe American Association of
Clinical Chemistry: New Orleans, USA. Contact AACC.
National Offices, 1752 K Street N.W., Suite 1010,
Washington, D.C. 20006.
Conference organizers should sendfull details oftheir meetingfor
inclusion in Journal ofAutomatic Chemistry's' calendar at least
three months before the event. Information to the Editor, Journalof
Automatic Chemistry, Taylor & Francis Ltd, 4 John Street,
London WC1N 2ET.
215

				

